# The Pharmacokinetic Changes in Cystic Fibrosis Patients Population: Narrative Review

**DOI:** 10.3390/medicines12010001

**Published:** 2024-12-31

**Authors:** Ayda Awaness, Rania Elkeeb, Sepehr Afshari, Eman Atef

**Affiliations:** Pharmacy School, West Coast University, Los Angeles, CA 90004, USA; aawaness@westcoastuniversity.edu (A.A.); relkeeb@westcoastuniversity.edu (R.E.); safshari1@u.westcoastuniversity.edu (S.A.)

**Keywords:** pharmacokinetics, cystic fibrosis, special population, gastrointestinal tract, absorption, pH, residence time, protein binding, volume of distribution, transmembrane conductance regulator (CFTR), drug–drug interaction

## Abstract

Cystic fibrosis (CF) is a rare genetic disorder commonly affecting multiple organs such as the lungs, pancreas, liver, kidney, and intestine. Our search focuses on the pathophysiological changes that affect the drugs’ absorption, distribution, metabolism, and excretion (ADME). This review aims to identify the ADME data that compares the pharmacokinetics (PK) of different drugs in CF and healthy subjects. The published data highlight multiple factors that affect absorption, such as the bile salt precipitation and the gastrointestinal pH. Changes in CF patients’ protein binding and body composition affected the drug distribution. The paper also discusses the factors affecting metabolism and renal elimination, such as drug–protein binding and metabolizing enzyme capacity. The majority of CF patients are on multidrug therapy, which increases the risk of drug–drug interactions (DDI). This is particularly true for those receiving the newly developed transmembrane conductance regulator (CFTR), as they are at a higher risk for CYP-related DDI. Our research highlights the importance of meticulously evaluating PK variations and DDIs in drug development and the therapeutic management of CF patients.

## 1. Introduction

Cystic fibrosis is a life-shortening genetic disease. Most patients are of Caucasian race, with an incidence rate of approximately 1 in 2000 live births [1]. However, it is notably less prevalent among Asian and African Americans populations [2].

The 2021 Cystic Fibrosis Foundation registry reports a rise in cystic fibrosis (CF) prevalence over the past decade. The latest estimate reporting nearly 40,000 individuals in the United States and 105,000 globally have been diagnosed with CF [3]. The median expected survival age for CF patients in the USA is 47.4 years (95% CI, 44.2–50.3) [4].

A mutation in both copies of the cystic fibrosis transmembrane conductance regulator protein (CFTR) gene causes this disease. The gene is on chromosome 7, which encodes the chloride channel. These mutations impair chloride and ion transport, leading to thick secretions in multiple organs, including the lungs, pancreas, liver, intestines, and reproductive system, resulting in a multisystem disease. There are five classes of CFTR mutations as listed in (Table 1) [5,6], and characterizing these mutations is crucial for guiding initial therapy, as recent treatments have been developed to target specific mutation types [4,5,7]. Before the approval of the first CFTR modulators in 2012, disease management relied exclusively on symptom-based treatments, such as clearing lung secretions and infections prophylaxes [8,9]. However, recent advances in treatment aim to target the underlying causes of the disease.

Due to the complex and varied degrees of pathophysiological effects of CF, the PK of many drugs may differ in CF patients compared to the general population. Thus, it becomes critical to consider these variations to optimize therapy and ensure effective and safe drug in this special population [1].

## 2. Literature Search

An electronic search on multiple databases, including PubMed, Scopus, and Google Scholar, was conducted to evaluate the primary literature and reviewed articles on CF PK studies published in English through 31 December 2023. We updated the search through October 2024. The following keywords were used: cystic fibrosis, pathophysiology, absorption, distribution, elimination, metabolism, excretion, gastrointestinal tract, pH, residence time, protein binding, volume of distribution, transmembrane conductance regulator (CFTR), drug–drug interaction, and CFTR modulators.

**Figure 1:**
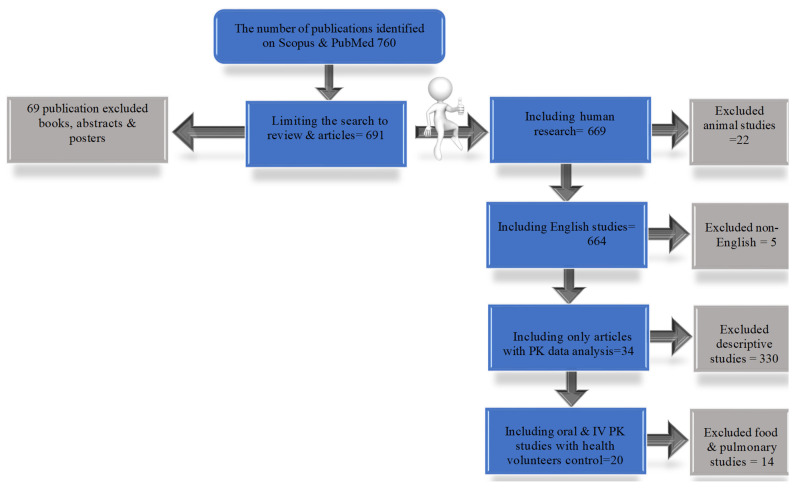
Literature search with excluded and included study numbers of PK in CF.

Current practice guidelines were reviewed, and we inspected reference lists of selected articles for additional relevant sources, as seen in Figure 1.

The search included English, oral and IV, and human studies that compared CF patients to healthy subjects. The animal and pulmonary studies as well as studies that compared PK of different routes of administrations in CF patients and did not include healthy subjects were excluded.

## 3. Pharmacokinetic Changes

### 3.1. Absorption

Many factors can affect drug absorption in CF patients, including pH, resident time, membrane permeability, and bile salts [1,10,11,12,13,14,15,16]. These factors can lead to diverse effects on the total drug absorption in CF patients when compared to healthy volunteers. (Table 2A).

#### 3.1.1. pH

Gastrointestinal pH plays a critical role in oral drug absorption and bioavailability by influencing drug dissolution, solubility, release, stability, and intestinal permeability. In cystic fibrosis (CF) patients, altered pH levels in the gastrointestinal tract, caused by factors such as reduced bicarbonate secretion and pancreatic insufficiency, can significantly affect drug solubility and absorption rates, potentially impacting therapeutic outcomes [38]. However, a study by Youngberg et al. comparing gastric pH in ten CF patients and ten healthy volunteers under fasting and postprandial conditions found that fasting gastric pH was similar between CF patients and healthy individuals, indicating normal gastric acid function in CF patients. Postprandial gastric acid secretion, in both groups, lowered the pH below 3 within 60 min, returning to a baseline of 2 or lower by the second hour [12].

The range of duodenal pH for a healthy population is 6–7.4 [10]; however, CF duodenal pH was 3–6 in a fed period. The mean CF jejunal pH of 6.2 was marginally lower than healthy volunteers of 6.6 [11]. Another study confirmed these results that showed a marginal drop in duodenal pH during the fed state when compared to healthy volunteers [12]. Duodenal hyperacidity is related to CFTR mutation. Defective CFTR leads to lower chloride ions, bicarbonate ions, and water secretions. The low concentrations of these ions lead to failure in neutralizing the hyperacidity of the duodenal content, thus altering the absorption of weakly ionized drugs [39].

Even though the studies have somehow contradicting findings. It is generally accepted that while CF patients may exhibit altered intestinal pH, their gastric acid function remains relatively normal under fasting conditions. This may affect drug solubility and dissolution in the intestine, but the more profound effect is on the release of drug from a pH-dependent enteric-coated formulation. This is especially important in terms of drug on-set of action.

#### 3.1.2. Residence Time

Altered residence time may influence drug absorption rates in CF patients. Several factors impact the residence time of drugs in the gastrointestinal tract, such as intestinal dysmotility or increased mucus production. Research indicates that fasting CF patients exhibit longer oro-rectal transit times than their healthy counterparts. This finding is supported by Gilbert et al., who reported that CF patients experience significantly extended intestinal residence times at pH levels below 6 during the first hour compared to healthy volunteers [17].

It was found that the passage through the small intestine is often significantly delayed in CF patients while the upper intestinal transit time is more rapid. However, no significant differences were found in median gastric emptying time [18]. The extended intestinal transit time could be due to multiple defective epithelial CFTR genes in the gastrointestinal tract acting as the primary insult. This defective CFTR causes chloride and water transport impairment across the apical membrane of the epithelial cells of the guts, leading to implications in the production of the thickened mucus [40]. Moreover, Murphy et al. explained that intestinal dysmotility could be due to increased gut hormones, motilin, entero-glucagon, neurotensin, and polypeptides YY. These hormones have a crucial role in gastrointestinal motor activity [41].

The findings suggest that, depending on the physicochemical properties of the drugs and the normal mechanisms of drug transport, the absorption profiles of drugs in CF patients may differ, regardless of whether there is a change in bioavailability.

#### 3.1.3. Membrane Permeability

The CFTR gene dysfunction leads to changes in intestinal membrane properties including permeability subsequently impacting the drug absorption. This can result in variable drug absorption profiles in CF patients compared to healthy individuals [42]. The small intestinal permeability in CF patients is 4–10 times greater than typical values [11]. Stead et al. conducted a PK study on ethinyl estradiol in CF patients. The oral bioavailability for CF patients was found to be significantly higher 76.9% (SD 11.7%) compared to the control 47.3% (SD 7.5%), which might be due to reduced conjugation of ethinyl estradiol in the gut wall of CF patients (Table 2A) [13].

In another study, the absorption from the upper small intestine of a combination of disaccharide lactulose and monosaccharide rhamnose was studied in CF children. The increased permeability could be attributed to the dysfunction of the intestinal epithelial layer due to changes to the tight junctions that compromises epithelial integrity [19].

Most CF patients exhibit exocrine pancreatic insufficiency leading to poor fat absorption and thus require pancreatic enzyme supplementation. Beringer et al. found that the bioavailability and rate of absorption of azithromycin were not altered in CF patients administering pancreatic enzyme compared to healthy subjects (Table 2A). The lack of a difference suggests that either absorption is unaffected by pancreatic insufficiency or supplementation by administration of exogenous enzymes was able to overcome the insufficiency [20]. Thus, the data are not definitive, as the study lacks a third group of CF patients not receiving pancreatic enzymes, which limits the scope of the findings (Table 2A).

#### 3.1.4. Bile Salts Deficiency

Bile salt Deficiency is common in CF patients due to pancreatic insufficiency. Bile salts play a crucial role in the solubilization of lipophilic drugs and thus their absorption is impacted in CF patients [43].

Bile acid salts precipitate at a rate three times higher in CF patients compared to healthy individuals. As a result, their emulsification function is impaired, leading to increased loss of bile acids in the feces [23].

Absorption is dependent on several factors, including the presence of pancreatic enzymes and bile salts. In one study, CF patients received retinal, a fat-soluble vitamin, with enzyme supplements to eliminate the effect of enzyme deficiency. The study showed a significantly lower retinol median plasma concentration in CF patients, possibly due to the bile acids malabsorption [44].

One of the factors that influences absorption is the availability of bile salts. In one study, CF patients received retinol, a fat-soluble vitamin, and enzyme supplements to mitigate the enzyme deficiency factor [14]. The CF patients had significantly lower median plasma retinol concentrations, attributed to bile acid malabsorption [44].

In conclusion, the pathophysiology of CF patients can alter drug absorption due to various factors, including the physicochemical properties of the drug, gastrointestinal pH, transit time, motility, co-administered supplements, and bile acid insufficiency. The severity of these changes depends on the stage of the disease. Therefore, individualized evaluation of each drug, particularly those with narrow therapeutic indexes, is essential [45].

### 3.2. Distribution

We studied the possible factors that could affect drug distribution in CF patients. The two main factors investigated in the literature are protein binding and body composition [22] (Table 2B). It is reported that CF patients may experience hypoalbuminemia, potentially affecting the free fraction of drugs with pronounced clinical effects on highly albumin-bound drugs. The common causes of hypoalbuminemia in CF may include increased plasma volume due to pulmonary hypertension or a reduced rate of protein synthesis resulting from advanced hepatic cirrhosis [22].

In one study that investigated two high albumin-bound β-lactams, it was reported that CF patients had an up to 2.0, 1.4-fold increase in unbound fractions for dicloxacillin and cloxacillin, respectively. This could be explained by a decreased plasma protein binding for these hydrophilic β-lactams antibiotics [24]. It was also found that 42% of CF patients had albumin concentrations more than two standard deviations below those of the control group. In cases where albumin levels are significantly lower than normal, it is critical to adjust the dosage of narrow therapeutic index drugs based on the patient plasma free fraction [25].

Another less commonly studied protein, yet critical for CF patients, is retinol-binding protein (RBP), a marker for retinol bioavailability. CF patients are at risk for vitamin A (retinol) deficiency due to fat malabsorption, therefore; retinol is among the common vitamins administered to CF patients [46]. Retinol binds to retinol-binding protein at a 1:1 molar ratio [26]. Twelve percent of CF patients had significantly lower RBP than the control group, which explains the low bioavailability of retinol in CF patients [26]. Thus, adjusting the retinol level is crucial to mitigate the risk of hypervitaminosis [47].

The observed differences in the drug volume of distribution (Vd) per unit weight can also be attributed to the changes in body weight and composition. Due to the altered body composition in CF patients, leading to a decline in adipose tissue, lipophilic drugs distribution will drop compared to healthy individuals. As a result, these drugs may remain in the bloodstream at higher concentrations, leading to a lower apparent Vd than expected. This will affect dosing regimens and possibly the drug toxicities. On the other hand, the hydrophilic drug Vd is expected to increase due to the increased lean tissue per kg body weight of CF patients. Another important factor to consider is body composition. The body mass index (BMI) is a generally accepted measure for assessing the CF patients’ weight and nutritional status. However, BMI does not distinguish between body compartments such the adipose tissues, fat-free mass (FFM), lean body mass (LBM), and total body water. The ratio between these tissues is different in CF patients. It was found that the difference in Vd differences of most drugs vanished when corrected for lean body mass [48].

### 3.3. Elimination

#### 3.3.1. Renal Clearance

Multiple studies examined renal drug clearance in CF patients. Many focused on antibiotics as a commonly prescribed drug for CF patients. The published studies included ciprofloxacin, ceftazidime, dicloxacillin, pefloxacin, dicloxacillin, and probenecid [21,27,30,32,49] (Table 2C).

In two independent studies, the renal clearance of ciprofloxacin in CF patients was not statistically different compared to healthy volunteers [27,28]. One study suggested that the increase in renal elimination is compensated by a decrease in Vd, resulting in an overall CF renal clearance comparable to that observed in healthy volunteers [50].

Pefloxacin is a lipophilic quinolone predominantly non-renally cleared. Pefloxacin renal clearance was approximately 53% larger in CF patients, yet the bioavailability and disposition were comparable between the CF and healthy groups as the nonrenal clearance accounted for approximately 90% of total pefloxacin clearance. The CF urinary excretion of pefloxacin metabolites was only slightly higher in patients with CF [21].

Bulitta et al. reported a 19% reduction in clearance and a 36% decrease in the volume of distribution for ceftazidime in patients with cystic fibrosis (CF) compared to healthy volunteers. However, the study did not provide statistical data to confirm the significance of these findings, so the reported 19% reduction in clearance may or may not be statistically significant (Table 2C) [49]. Contrary to the above finding, Hedman et al. reported a significant increase in average renal clearance of ceftazidime in CF patients (mean ± SD: 125 ± 20 mL/min/1.73 m^2^) compared to healthy controls (100 ± 9 mL/min/1.73 m^2^). The same group also studied the renal clearance of CF patients, reporting an increased inulin clearance (132 ± 30 mL/min/1.73 m^2^) compared to healthy controls (103 ± 8 mL/min/1.73 m^2^). These results indicate a higher, yet more variable, glomerular filtration rate in CF patients (ranging from 74 to 174 mL/min/1.73 m^2^), leading to increased and variable elimination of ceftazidime and possibly other renally eliminated antibiotics. This finding was also confirmed by Jusko et al. They also reported a 55% creatinine clearance elevation in the CF patients [30]. The ceftazidime PK variabilities are not clinically significant and may not warrant dosage adjustments in CF patients such as in the case of ceftazidime [29]. Two PK studies investigate the PK changes of dicloxacillin in CF patients. While Jusko et al. reported remarkably high renal clearances of dicloxacillin which average (282 ± 135 mL/min/1.73 m^2^) in CF patients compared to (95 ± 28 mL/min/1.73 m^2^) in healthy subjects [30], Beringer et al. concluded no significant difference in the renal clearance of dicloxacillin in patients with cystic fibrosis compared to healthy volunteers [32]. The challenge in dicloxacillin renal elimination studies is the multiple clearance mechanisms of the drug. In addition to the increased glomerular filtration rate, Susanto et al. concluded that P-glycoprotein (P-gp) substrates antibiotics renal clearance is higher in CF patients due to increased P-gp expression leading to active secretion. The same group reported that antibiotics that are not P-gp substrates, such as cefsulodin and sulfamethoxazole, did not exhibit increased clearance in CF patients [50].

The sulfamethoxazole finding agreed with another independent study by Hutabarat et al. [34].

Fleroxacin PK in CF patients showed a significant increase in renal clearances of fleroxacin and its metabolites. This was explained by the decreased tubular reabsorption of these compounds [31]. The PK of tobramycin in CF patients showed a higher tobramycin total body clearance (121.2 ± 14.2 mL/min/1.73 m^2^) than did controls (102.2 ± 18.9 mL/min/1.73 m^2^, *p* < 0.05). Tobramycin renal clearance was not significantly different in the two groups. The increased tobramycin total body clearance without an increase in renal clearance indicates that an extrarenal clearance pathway might play a significant role in the elimination of tobramycin from the serum in CF patients [33].

The same observation was confirmed by Kearns et al. when studying the PK of gentamicin, another aminoglycoside in children and young adults with CF. The study revealed a significantly larger total plasma clearance for CF patients [51].

In summary, the increased renal elimination of some antibiotics in CF patients could be attributed to a higher activity of the renal P-glycoprotein, increased active secretion or increased glomerular filtration rate.

#### 3.3.2. Hepatic Metabolism

Changes in liver enzyme activity are the main reason behind the increased hepatic clearance of drugs in CF patients. In addition to disease effect, some studies also investigated the enzymes genetic variability [52]. Patients with CF have enhanced clearance of drugs metabolized by CYP1A2 and CYP2C8, which showed increased activity in CF patients, whereas drug metabolism by both CYP2C9 and CYP3A4 is unaffected [53].

Fleroxacin is a fluoroquinolone and a substrate of CYP1A2, is rapidly and completely absorbed and extensively metabolized. When normalized for lean body mass, the metabolism and clearance of fleroxacin major metabolites were significantly higher in CF patients [31]. These data concur with later studies showing a significant increase in cystic fibrosis metabolism. The two major metabolites’ formation and elimination were 40% to 70% higher in patients with CF [35] (Table 2D).

Hutabarat et al. studied both the disposition of sulfamethoxazole and trimethoprim and acetaminophen [34,36]. Sulfamethoxazole and trimethoprim were investigated in adult CF patients. The total plasma clearance of sulfamethoxazole increased in CF patients (26.2 ± 6.4 mL/h/kg) compared to the control subjects (18.8 ± 4.3 mL/h/kg). This increase was mainly attributable to increased metabolic clearance of sulfamethoxazole with the renal clearance of sulfamethoxazole remaining unchanged [34]. The same group studies the disposition of oral acetaminophen in CF adults. The total plasma clearance of acetaminophen was higher in CF patients (362 ± 81 mL/h/kg) than in control subjects (247 ± 22 mL/h./kg). This difference in clearance was also attributable to a greater metabolic clearance of acetaminophen to acetaminophen sulfate and glucuronide [36]. Montelukast, a drug mainly metabolized by CYP2C9 and CYP3A4. Its elimination involves hepatic metabolism and biliary excretion. A study that compared the PK of the montelukast in CF and healthy volunteers showed a higher but insignificant AUC in CF patients. The clearance of the CF patients was 55.53 ± 43.95 mL/min, compared to 57.12 ± 18.42 mL/min in healthy volunteers, thus concluding no significant difference in clearance [54]. Ciprofloxacin, an antibiotic mainly metabolized by CYP1A2, showed an increase in hepatic clearance, as reported by Christenson et al. [55], in contrast to this finding, Kennedy et al. reported that CYP1A2 did not show an increase in hepatic clearance this could be due to limitation of the study conducted on children with mild CF symptoms only [56].

The PK of theophylline, another CYP1A2 substrate, was compared between CF and healthy volunteers. The CF patients’ total body clearance was twice that of the control subjects (*p* < 0.001). The volume of distribution of theophylline was also greater in the patients with CF (*p* < 0.05), which may have contributed to the increased clearance. This suggests that a larger dose of theophylline may be needed if an asthmatic CF patient needs it [57].

Another study assessed the intravenous lidocaine metabolism in CF patients [37]. The results showed that CF patients had significantly reduced monoethylglycinexylidide levels compared to controls, indicating impaired lidocaine metabolism (39.4 ± 16.9 μg/L vs. 70.3 ± 45.7 μg/L). The monoethylglycinexylidide is a metabolite of lidocaine by CYP3A4 isoform. This could be possibly attributed to decreased hepatic activity of the CYP3A4 in CF patients [37]. Pharmacokinetic parameters of ethinyl estradiol were compared between women with CF and a control group. The findings demonstrated that total body clearance of ethinyl estradiol was significantly increased in CF patients compared to the control group. However, this was offset by a higher bioavailability of the drug in the CF group resulting in comparable area under the curve in both groups [13].

It is worth mentioning that ethinyl estradiol self-induces its own metabolism via CYP1B1, causing an increased metabolism and elimination from the body [58].

Many studies reported the CF enhanced activity of CYP1A2, and reduced activity of the CYP3A4.

Some studies reported an increased biliary elimination, while few others discussed the potential reduction of biliary elimination [59].

## 4. Drug–Drug Interactions

Patients with cystic fibrosis (CF) are anticipated to experience substantial improvements in lung function and nutritional status following the development of the CFTR modulators. The CFTR modulators are small molecules that improve the mutated CFTR protein’s functions through various mechanisms, including potentiators, which improve the channel gating; correctors, which facilitate the movement of CFTR through cell membranes; stabilizers, which prolong the presence CFTR at the cell membrane; amplifiers, which increase the CFTR quantity; and readthrough agents, which suppress premature termination codons (PTCs) within the genetic code [8].

**Figure 2:**
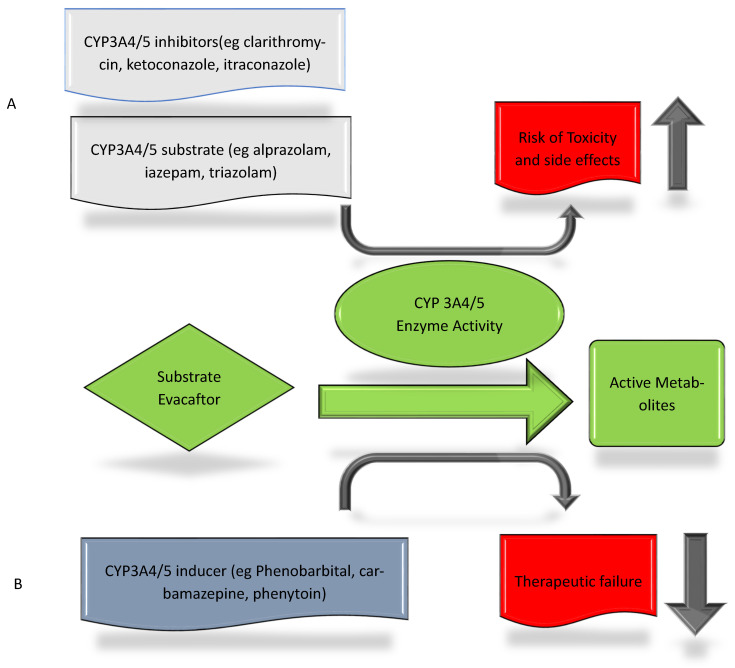
(**A**) Effects of CYP3A4/5 enzyme substrates and inhibitors on ivacaftor, a CYP3A4/5 enzyme metabolizer. (**B**) Effects of CYP3A4/5 enzyme inducers on ivacaftor, a CYP3A4/5 enzyme metabolizer.

The first triple combination of CFTR modulators was approved in 2019 (elexacaftor–tezacaftor–ivacaftor tablet). The drug combination has a potential for drug–drug interactions (DDI) related to CYP enzymes and P-gp. The liver CYP enzymes, mostly CYP3A4/5, extensively metabolize the three components [60,61]. Thus, concomitant administration of these drugs with CYP3A4/5 substrate, inducer, or inhibitor will alter the CFTR modulators serum concentration [60,62,63,64]. CYP3A4 substrates that may interact with ivacaftor include alprazolam, diazepam, midazolam, triazolam, and tacrolimus. Co-administration of these drugs with ivacaftor may elevate their serum concentrations (Table 2, Figure 2). Moreover, combining ivacaftor with warfarin, a CYP2C9 substrate, might increase exposure to warfarin since ivacaftor is a CYP2C9 inhibitor [61]. Concomitant administration of ivacaftor, P-gp inhibitor, with digoxin a P-gp substrate, increases exposure to digoxin by 1.3-fold. The latter can be a potential DDI with many P-gp substrates.

Also, concomitant administration of ivacaftor with strong CYP3A4 inducers decreases the ivacaftor effects due to induction of the drug metabolism [63] (Figure 2).

The concomitant use of (lumacaftor–ivacaftor) tablets with itraconazole, a strong CYP3A4 inhibitor, increases ivacaftor exposure by 4.3-fold; however, it did not impact the exposure of lumacaftor. As the lumacaftor is a CYP3A4 inducer and its effect will offset the CYP3A4 enzyme inhibition effect of itraconazole after reaching a steady state of (lumacaftor–ivacaftor) tablets with itraconazole usage, no dose adjustment for (lumacaftor–ivacaftor) is needed [64].

When co-administered with CYP3A4 inhibitors, the levels of elexacaftor, tezacaftor, and ivacaftor increase up to 2.8-fold, 4.5-fold, and 15.6-fold, respectively. As a result, it is advised to reduce the dose of elexacaftor–tezacaftor–ivacaftor when combined with moderate or strong CYP3A inhibitors [60,62] (Table 2, Figure 2).

## 5. Challenges in CF Pharmacokinetic Studies

Cystic fibrosis PK studies present several challenges, including the complex and varied pathophysiological changes and severity that can influence absorption, distribution, metabolism, and excretion processes. Furthermore, ethical considerations arise when including CF patients in PK studies. Especially the ones that are not included in their current treatment regimen or require discontinuation of their current administered medications.

Other challenges include a limited patient population and reduced life expectancy, particularly among those with more severe protein defects that exacerbate the disease’s severity.

Cystic fibrosis results from different underlying genetic variability; thus, researchers should investigate and document the pharmacogenomics of these patients [65,66].

Due to accessibility and cost considerations, future variability will arise between CF patients with access to CFTR modulators and those without.

## 6. Conclusions

Recognizing CF patients as a special PK population is essential for implementing dose adjustments that ensure drug safety and efficacy. The CF population experiences significant pathophysiological changes affecting absorption, distribution, metabolism, and excretion. Consequently, developing a CF PK population model is vital for initial drug dose adjustment and possible DDI prediction. This approach, combined with therapeutic drug monitoring, helps to ensure optimal outcomes, particularly for patients with advanced disease progression.

It is important to consider factors such as intrasubject variability, the disease’s stage, and the specific genetic mutation of CF. Furthermore, drug–drug interactions must be carefully evaluated, especially given the multitude of drugs that patients may be taking concurrently, including new CFTR modulators, which can lead to various pharmacokinetic interactions.

## Figures and Tables

**Table 1:** Pathophysiology and classification of CF mutation classes and their effects [5,6].

Class	Description
Class 1: Protein production mutations	Lead to the production of a defective protein and is usually caused by nonsense, frameshift, or splice site mutations. This leads to premature termination of mRNA transcripts and a complete absence of CFTR protein.
Class 2: Protein processing mutations	Involve a defective protein being processed, causing abnormal post-translational processing of the CFTR protein. This prevents the protein from reaching the correct cellular location.
Class 3: Gating mutations	The CFTR protein acts as a gate to allow for chloride passage, but it remains closed when not in use. Gating mutations can hinder the gate from fully opening, which limits or prevents chloride from entering the cell.
Class 4: Conduction mutations	They cause changes in one or more amino acids forming the CFTR protein deterring the CFTR function, even though the protein shape is maintained. This interferes with the smooth movement of chloride through the channel.
Class 5 Insufficient protein mutations	insufficient production of CFTR by the body, partial functionality of CFTR, or rapid breakdown of the protein at the cell’s surface.

**Table 2:** Comparing PK parameters in CF vs. healthy volunteers.

**A. Absorption Factors**											
1. pH changes across GI (stomach, small intestine, and terminal ileum)		CF					HV			* Statistics	Ref.
		Preprandial		Postprandial			Preprandial	Postprandial			
Gastric		pH < 1–4.7		pH: 3–2 after 60 min			pH < 1–3.2				[12]
Duodenal		pH: 6		pH: 3–6			pH: 5–6	pH: 6–7.4		*p* < 0.02 in CF Patients	[10,12]
Jejunum		pH > 6.0		pH: 5.75–6.5 (90 min) pH: 6.2 (3 h)			pH:6.5–6.8 Mean pH: 6.6	4.8–6.3 (3 h) Mean pH: 5.6			[11,12]
Total terminal ileum		pH: 5.5–7.2						pH: 7.5			[11]
2. Gastrointestinal transit times and motility		CF					HV				Ref.
Gastric emptying time		Median gastric emptying time was 58 min (range 6–107)					Median gastric emptying time was 41 min (range 4–125)				[17,18]
Orocecal transit time		(0.8–6.5) h at 7 h, 88% of the tracer reached the cecum					(0.5–2.5) h at 7 h, 20 of the tracer reached the cecum				
3. Membrane permeability		CF					HV			* Statistics	Ref.
Drugs Bioavailability											
Ethinyl estradiol %		76.9% (SD 11.7%)					47.3% (SD 7.5%)			*p* < 0.001	[13]
Lactulose–rhamnose extraction ratio		0.16 ± 0.022					0.038 ± 0.003			Mean ± SEM	[19]
Azithromycin %		34.2%					42.8%			*p* = 0.37	[20]
Retinol		298 × 10^−5^					458 × 10^−5^			*p* < 0.01 compared with controls	[14]
Cloxacillin		50.2 ± 26.2%					38.4 ± 16.7%				[19]
Pefloxacin		107% (58–178)					99% (80–141)				[21]
4. Bile acids output and excretion		CF					HV			Significant difference pH < 5 vs. pH > 6	
Aqueous bile acids		pH < 5	pH 5–6			pH > 6	15.1–272.8 (mmol/L)			*p* < 0.001	[11,22]
		4.7 ± 0.9 (mmol/L)	9.0 ± 1.5 (mmol/L)			12.5 ± 2.0 (mmol/L)					
Bile acid precipitation (%)		45.7 ± 5	18.2 ± 4			14.5 ± 8	3–5%			*p* < 0.001	[23]
Fecal Bile acid excretion		21.5 (2.4) (µmol/kg/24 h)					7.3 (1.2) (µmol/kg/24 h)				[23]
**B. Distribution Factors**											
Protein Binding		Fraction bound in Plasma					Fraction unbound (fu) in plasma			Ratio	Ref.
		CF			HV		CF		HV	fuCF/fuHV	
Dicloxacillin		88.4 ± 7.7%			94.4 ± 1.9%		11.6 ± 7.7%		5.6 ± 1.9%	2.07	[24]
Cloxacillin		94.8 ± 5.1%			96.2 ± 2.1%		5.2 ± 5.1%		3.8 ± 2.1%	1.37	[24]
Protein concentration		CF					HV			* Statistics	Ref.
Albumin conc		41.9 ± 5.4 g/L					45.8 ± 2.1 g/L			4–8 SD below normal	[25]
Retinol-binding protein (RBP)		28.9 ± 8.3 mg/L					36.0 ± 7.8 mg/L			Significant difference	[26]
Retinol: RBP		1.27 ± 0.16 mol/mol					1.43 ± 0.17 mol/mol			Significant difference	[26]
Volume of distribution											
Ethinyl estradiol		4.84 (3.00) L/kg					3.66 (1.13) L/kg			No significant difference	[13]
**C. Renal Clearance**											
Drugs Explored		CF					HV			Statistics	Ref.
Ciprofloxacin IV 200 mg		17.9 ± 6.93 (L/h)					20.8 ± 3.67 (L/h)			No significant difference	[27]
Ciprofloxacin 500 mg oral dose		474.1 ± 159.5 (mL/ min)					395.6 ± 139.0 (mL/min)				[28]
Ceftazidime		125 ± 20 mL/min/1.73 m^2^					100 ± 9 mL/min/1.73 m^2^			25% Increase	[29]
Dicloxacillin		282 ± 135 mL/min/1.73 m^2^					95 ± 28 mL/min/1.73 m^2^			Significant difference	[30]
Fleroxacin		44.8 ± 12.8 mL/min/50 kg LBM					40.9 ± 10.7 mL/min/50 kg LBM				[31]
Dicloxacillin + probenecid		1.7 (L/h/1.73 m^2^)					1.9 (L/h/1.73 m^2^)			Probenecid delays the elimination of dicloxacillin	[32]
Tobramycin		121.2 ± 14.2 mL/min/1.73 m^2^					102.2 ± 18.9 mL/min/1.73 m^2^			*p* < 0.05	[33]
**D** **. Non-Renal Elimination (Hepatic Metabolism)**											
Hepatic Metabolism	CYP enzymes	CF					HV			* Statistics	Ref.
Sulfamethoxazole and trimethoprim clearance	CYP3A4, CYP2C9, and to lesser extent CYP1A2	26.2 ± 6.4 mL/h/kg					18.8 ± 4.3 mL/h/kg				[34]
Acetaminophen clearance	CYP1A2, CYP3A4, and CYP2E1	362 ± 81 mL/h/kg					247 ± 22 mL/h/kg			*p* < 0.025	[35]
Ethinyl estradiol clearance	CYP3A4 and CYP2C9	0.61 ± 0.19 L/h/kg					0.32 ± 0.16 L/h/kg			*p* < 0.02	[13]
Montelukast clearance	CYP3A4, CYP2C8, and CYP2C9	55.53 ± 44.0 mL/min					57.12 ± 18.42 mL/min				[36]
Lidocaine t1/2	CYP1A2 and CYP3A4	4.6 ± 0.9 min					3.0 ± 0.3 min			*p* = 0.093	[37]
Azithromycin (elimination constant)	CYP3A4	0.693 h^−1^					0.492 h^−1^			*p* < 0.01	[20]

CF: cystic fibrosis patients; HV: healthy volunteers. * Statistics only indicated whenever published in the cited references.

## Data Availability

No new data were created or analyzed in this study. Data sharing is not applicable to this article.
